# Exploring the Effects of Sweet Potato Leaves on Skin Health—From Antimicrobial to Immunomodulator

**DOI:** 10.3390/molecules30040855

**Published:** 2025-02-13

**Authors:** Manuela Machado, Sara Silva, Manuela Pintado, Eduardo M. Costa

**Affiliations:** Universidade Católica Portuguesa, CBQF Centro de Biotecnologia e Química Fina-Laboratório Associado, Escola Superior de Biotecnologia, Rua Diogo Botelho 1327, 4169-005 Porto, Portugal; mmachado@ucp.pt (M.M.); mpintado@ucp.pt (M.P.)

**Keywords:** sweet potato leaves, phenolic compounds, antimicrobial activity, biofilm inhibition, cytotoxicity, immunomodulation

## Abstract

Sweet potato leaves (SPL), an agricultural byproduct, hold significant potential in dermatological applications due to their bioactive compounds. This study evaluates the phenolic profile of SPL extracts and investigates their biological activities relevant to skin health. Extract fingerprinting, through uHPLC-DAD and LC–MS, identified phenolic acids and flavonoids, with cynarin, neochlorogenic acid, and spiraeoside being predominant. The presence of hyperoside was detected. From a biological standpoint, SPL demonstrated notable antimicrobial activity, with MICs ranging from 2.5 to 5 mg/mL against various bacterial strains, such as MRSA and *P. aeruginosa*, and effective antibiofilm activity, as it reduced biofilm formation by over 80% for most tested strains. When evaluating its effect upon keratinocytes, the cytotoxicity assessment revealed safe usage concentrations at 111 µg/mL and immunomodulatory capacities, as it increased IL-6 production in unchallenged cells but had no synergistic effects under inflammatory stimuli. While preliminary, and with more assays being necessary, these findings highlight SPL’s potential as a natural agent for antimicrobial and anti-inflammatory applications in skin-related applications and open a new avenue for a possible added value application of SPL.

## 1. Introduction

Skin health plays a key role in human well-being, as the skin is the body’s first line of defense against external threats and microbial colonization [1]. However, modern challenges, such as pollution and antimicrobial resistance, have led to a rise in the development of inflammatory skin diseases, such as atopic dermatitis and acne, and to premature skin ageing and a lack of efficacy of traditional solutions [2]. This, in conjunction with consumers, growing demand for natural and eco-friendly alternatives has led researchers to search for innovative, natural and sustainable solutions that can be used to protect and restore skin integrity [3]. Within this paradigm, plant-based byproducts, or plant byproducts based on bioactive compounds, are of particular relevance.

Sweet potato (*Iponmoea batatas* L) leaves (SPL), traditionally considered as an agricultural byproduct, are of particular interest in this regard due to their richness in bioactive compounds [4]. With the phenolic acids, flavonoids and vitamins contained in SPL, and the notable antimicrobial, anti-inflammatory properties ascribed to SPL, recent research efforts have focused on their use as nutraceuticals, particularly with respect to hepatoprotection, hypoglycemic and hypouricemic control, and diabetes modulation [5,6,7,8]. Despite this, and with a production of over 88.97 million tons in 2021, an estimated 95% of leaves are still being discarded, contributing to a significant waste of resources and waste production [5,9]. Within this paradigm, the possible use of SPL for dermatological care would be of great interest as it would open a new avenue of value for this byproduct while simultaneously promoting a sustainable solution for the tons of materials produced yearly.

SPL’s potential for skin-related applications is still unexplored. However, emerging data regarding SPL’s antimicrobial and anti-inflammatory potential and the biological potential associated with caffeic acid and quercetin (the main compounds associated with SPL), supports and lends credence to the use of SPL in skin-related applications [9,10]. In fact, as with other byproduct-based solutions, leveraging this underused resource may be a step towards enhancing the sustainable production of functional and natural skin health products. Taking this into consideration, this work aims to start building a knowledge base on SPL applications in skin by focusing on evaluating the extract’s antimicrobial and antibiofilm activities against skin commensal/pathogen microorganisms, followed by assessing the extract’s cytotoxicity and immunomodulatory effects upon skin keratinocytes. By integrating these aspects, this study aims to provide an insight into SPL’s potential in dermatological applications while contributing to the sustainable valorization of agricultural byproducts.

## 2. Results

### 2.1. Phenolic Compounds Fingerprint by uHPLC-DAD

**Figure 1:**
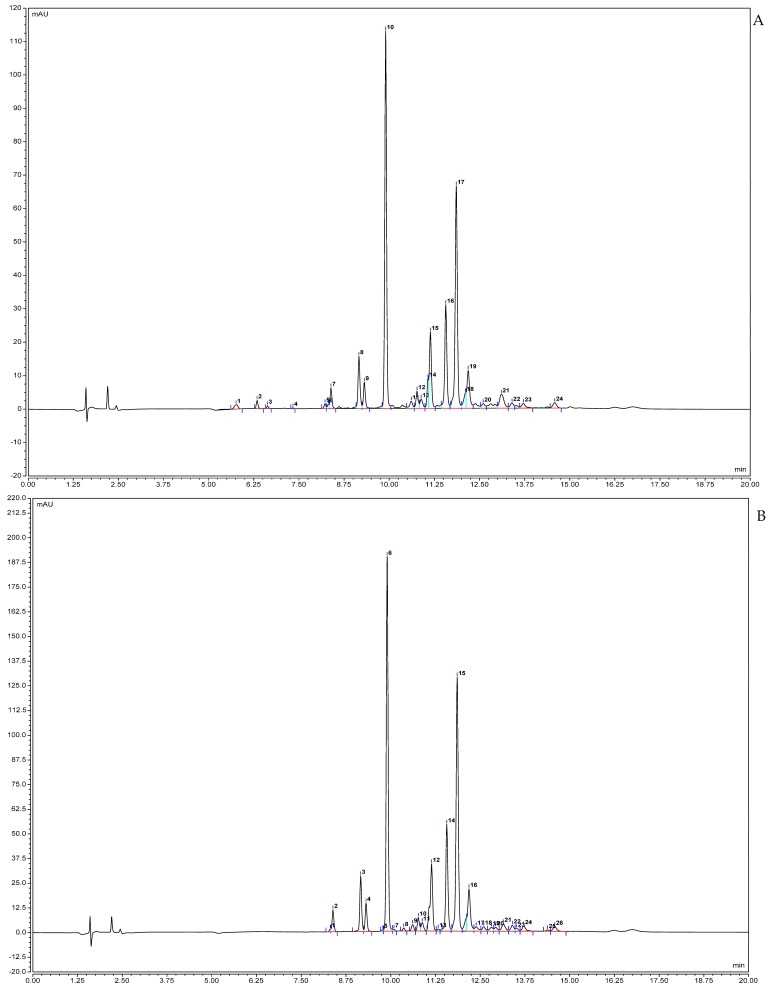
Phenolic compounds detected in the SPL extract by uHPLC-DAD fingerprinting: (**A**)—280 nm; and (**B**)—320 nm.

Regarding the compound fingerprinting of the SPL extract, one can see that at 280 nm (Figure 1A) 24 individual peaks were detected, while at 320 nm (Figure 1B) 26 individual peaks were detected. Through Area-Under-the-Curve (AUC) calculations and the use of a chlorogenic acid as a standardm it was possible to ascertain that the SPL extract evaluated had an average Total Phenolic Content (TPC) of 3.53 ± 0.0885 g Chlorogenic Acid Equivalents (CAE)/100 g of extract and an average Total Flavonol Content (TFC) of 3.13 ± 0.0744 g CAE/100 g of extract.

### 2.2. Phenolic Compounds Identification by LC–MS

**Figure 2:**
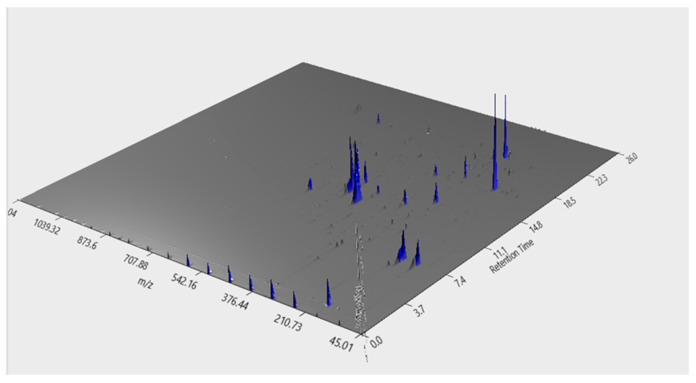
3D plot chromatogram of SPL extract visualized by MZmine.

Regarding the identification of individual compounds in the SPL extract, the data obtained are shown in Figure 2 and Table 1. In Figure 2, it is possible to see that the SPL extract is not overtly complex, with various low-intensity peaks that were not identifiable in the various databases assessed.

Nevertheless, as can be seen in Table 1, it was possible to identify 11 individual phenolics. Of the compounds identified, the following three were present at higher concentrations: cynarin (C_25_H_24_O_12_); neochlorogenic acid (C_16_H_18_O_9_); and spiraeoside (C_21_H_20_O_12_).

### 2.3. MIC and MBC Determination

Regarding SPL’s antimicrobial activity, the data obtained (Table 2) showed that a MIC value of 5 mg/mL was obtained for all microorganisms, with the exception of MRSA, for which a MIC of 2.5 mg/mL was registered. On the other hand, MBC values were only detected for *Pseudomonas aeruginosa* (*P. aeruginosa*) and MSSA. Interestingly, these were equal to the MICs detected for these two microorganisms.

### 2.4. Biofilm Formation Inhibition

**Figure 3:**
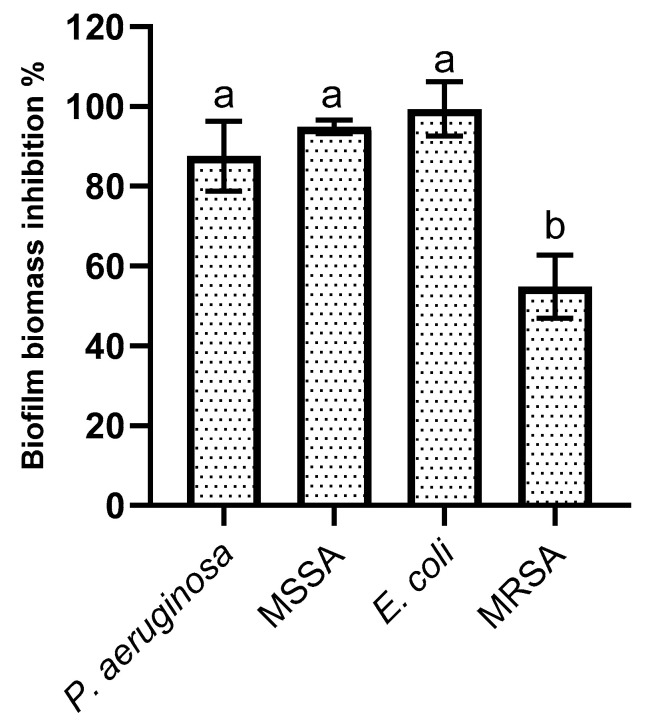
Biofilm formation inhibition values registered for the SPL extract at ½ of the MIC against the tested microorganisms. The letters above each bar indicate the statistically significant (*p* < 0.05) differences found for each microorganism.

When evaluating the effectiveness of the SPL extract upon the selected bacteria biofilm formation, the obtained data (Figure 3) showed that SPL reduced bacterial biofilm formation by over 80%, with the only exception being MRSA, for which a reduction of ca. 50% was observed. Among the data obtained, a statistically significant (*p* < 0.05) lower value was attained for MRSA compared to that of MSSA. A lack of statistically significant (*p* > 0.05) differences were observed between *P. aeruginosa*, *E. coli*, and MSSA.

### 2.5. SPL Extract Impact on HaCat Cells

**Figure 4:**
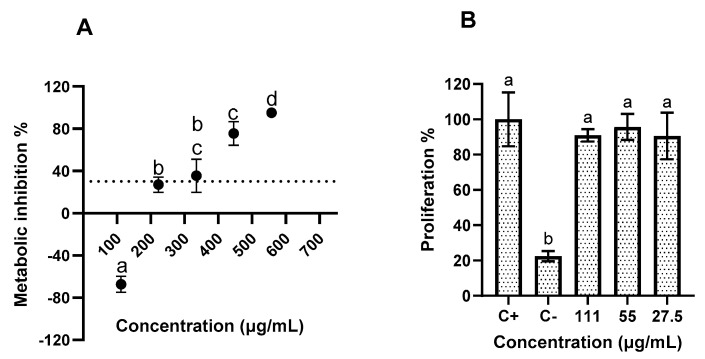
Evaluation of the impact of SPL extract on HaCat cell metabolism (**A**); and proliferation (**B**). Different letters represent the statistically significant (*p* < 0.05) differences found between conditions. The dotted line in (**A**) represents the 30% cytotoxicity limit, as defined by the ISO 10993-5:2009 Standard.

The data obtained regarding the impact of SPL extracts upon skin cell metabolism (Figure 4A) showed that, of the tested concentrations, only the lowest two concentrations were below the 30% inhibition limit, the value defined as safe by the ISO Standard 10993-5 [11]. Of the two concentrations below this value, the lowest tested concentration, 111 µg/mL, was selected for further assays, as the inhibition value of 27 ± 7.36% registered for the 222 µg/mL concentration was considered to be borderline cytotoxic. With respect to the SPL extract’s effect on HaCat’s cell proliferation activity (Figure 4B), the obtained data showed that SPL had no impact on HaCat’s proliferation at the tested concentrations, as no statistically significant differences (*p* > 0.05) were registered.

### 2.6. SPL Extract Impact on HaCat Cell Viability

**Figure 5:**
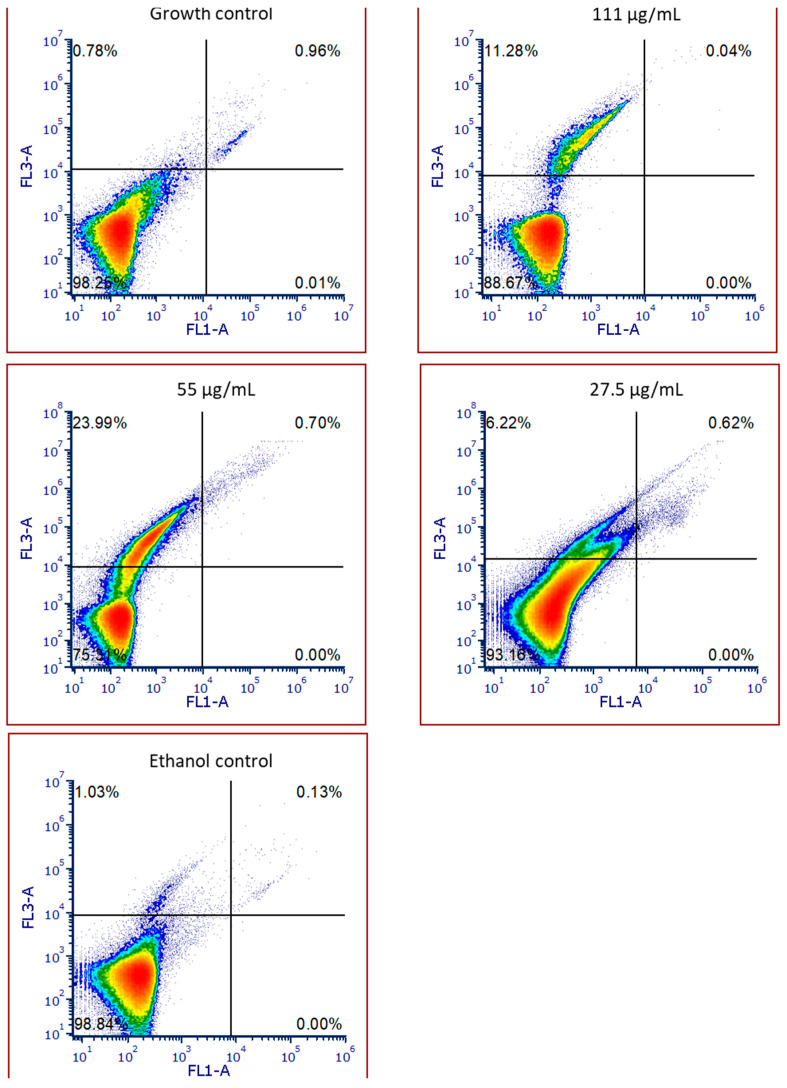
Live, early apoptotic, necrotic, and dead percentages of cells (%) after exposure to various concentrations of SPL extract and the EtOH control at 1% (*v*/*v*) on HaCat cells. Top left quadrant—necrotic/dead cells; Top right quadrant—late apoptotic cells; Bottom left quadrant—live cells; and Bottom right quadrant—early apoptotic cells.

The impact of the SPL extract on HaCat cell viability can be seen in Figure 5. When comparing the tested concentrations with the growth control condition (Figure 5), one can see the presence of necrotic/dead cells (top left quadrant) at all concentrations of SPL. Furthermore, when considering the data attained for the ethanol control (Figure 5), it is clear that this necrotic/dead population was not a consequence of the ethanol content of the extract. Interestingly, it is not possible to ascertain a dose–response pattern, as the highest necrotic/dead population (23.99%) was observed for the second-highest concentration tested (55 µg/mL) but not the first. Of the data obtained, it is notable that all tested concentrations fell within the safe limits of usage, as all were within the 30% cytotoxicity limit of the ISO Standard 10993-5 [11]. Regarding the prevalence of a necrotic/dead population over an apoptotic one in the presence of the SPL extract, this indicates the presence of an unplanned cellular death event, contrary to the normal apoptotic process, and is a secondary effect of SPL interaction with HaCat cells, due to the interaction of phenolic compounds with the cellular membrane causing irreparable cell damage, as previously described [12].

### 2.7. IL-6 Production by HaCat Cells

**Figure 6:**
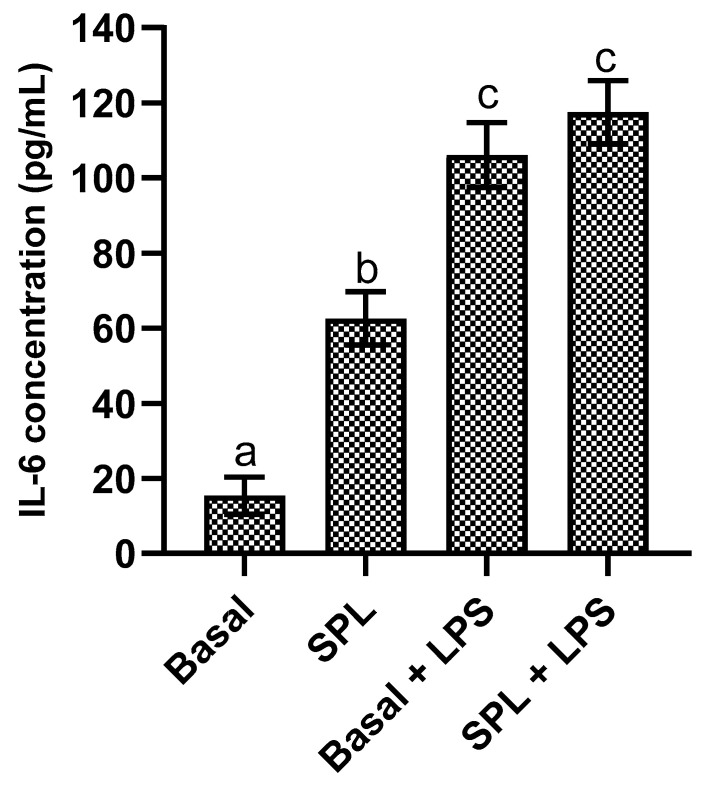
IL-6 production in pg/mL by HaCat cells in the presence of SPL extracts with and without inflammatory stimuli. Different letters represent the statistically significant (*p* < 0.05) differences found between conditions.

When evaluating the impact of SPL on IL-6 production in HaCat cells (Figure 6), it is possible to see that SPL on its own had a clear pro-inflammatory effect, as it increased the secretion of this interleukin by four-fold when in comparison with the basal control. On the other hand, when in the presence of a previous stimuli (LPS) SPL’s presence did not lead to significant increases in IL-6 secretion, and therefore did not show relevant synergistic effects.

## 3. Discussion

The TPC value reported in this work, 3.53 ± 0.0885 CAE/100 g of extract, is in line with that reported in previous works, as SPL ethanolic extracts have been shown to possess between 2.73 and 12.46 g CAE/100 g of extract [13]. A possible explanation for this value may be tissue age, as, according to Padda and Picha [14], TPC varies with tissue age, with younger sweet potato leaves presenting significantly higher values than mature or old ones such as the ones used in this work. With respect to the TFC value attained here, (3.13 ± 0.0744 CAE/100 g of extract) previous works do not provide a clear answer. On the one hand, Zhang, et al. [15] reported a TFC value of 56.87 mg rutin equivalents/g while on the other hand Fu, et al. [16] reported a TFC of 3.4 mg of quercetin equivalents/g of extract. Another factor that bears relevance is that previously, TFC has been reported as being roughly 50% of the TPC content, a value below the ca. 88% observed in this work [15].

When considering the individual compounds, previous fingerprinting and identification studies have reported values in line with those observed here; between 11 and 23 compounds have been detected in SPL leaves, with differences in compound quantity being associated with freeze-dried and fresh leaves [15,17]. Regarding the identified phenolic compounds, previous works support the data attained in this work. The main compounds usually attributed to SPL extracts are caffeic, chlorogenic, neochlorogenic, quercetin and its esters, and caffeoylquinic acids esters, either via HPLC or LC–MS, which have all been reported here [9,10,17,18]. Of the compounds identified in this work, three are of particular relevance. One is hyperoside, a flavonoid compound previously reported in SPL, which is commonly found in medicinal plants and is thought to contribute significantly to their medicinal potential [19,20]. The other is the identification of tiliroside, a natural glycosidic flavonol in plants with renowned anti-inflammatory activity and which has never been previously reported in SPL [21]. The last one is 3,5,5-Trimethyl-4beta-hydroxy-4-[3-(beta-D-glucopyranosyloxy)-1-butenyl]-2-cyclohexene-1-one, a compound that has not appeared in previous works.

When considering the antimicrobial potential of SPL, no previous works describe any MIC or MBC values for these extracts, as only inhibition halos have been reported. Of the existing reports, two reports indicated that SPL extracts have no activity against the bacteria used in this work [22,23] and one reported activity only against *E. coli* [18]. Considering these limitations, another possible analysis avenue is through the main constituents of SPL extracts. While their presence is not a guarantee of activity, as sometimes interactions between compounds may lead to reduced or even inhibited activities, they offer insights that may help to explain the results herein produced. Of the identified compounds, chlorogenic, caffeic and gallic acids have been previously reported as inhibiting MRSA, *S. aureus*, *P. aeruginosa* and *E. coli*, and may help to explain the activity observed here [24,25,26,27].

Regarding biofilm inhibition, no previous works exist regarding SPL extracts. When considering the SPL extract’s main compounds, previous works lend some support to the data obtained here, as *P. aeruginosa* and *S. aureus* were shown to be inhibited by sub-MIC concentrations of both chlorogenic and caffeic acid [24,25,28]. With respect to MRSA, previous works have reported varying levels of efficacy but reduced efficacy on this microorganism [24] (in line with what was observed here). Interestingly, both chlorogenic and caffeic acids were reported to possess reduced activity on *E. coli* and thus may not help to explain the data obtained here [28].

When considering SPL’s impact on HaCat cell metabolism, only one study showed that an aqueous SPL extract had no cytotoxicity towards HaCat cells up to 1 mg/mL, a value significantly superior to the one observed here [29]. When considering other cell lines, previous works follow the same pattern; aqueous SPL extracts were reported as not being cytotoxic to human mammary epithelial cells (MCF-10A) up to 400 µg/mL or to L929 cells (mouse fibroblasts) up to 1000 µg/mL [30,31].

Regarding cellular viability, previous works with SPL aqueous extracts showed that interactions with both MCF-10A and differentiated 3T3-L1 cells led to damage and to the appearance of a late apoptotic population [30,32]. These results are contrary to the one observed here, where the presence of SPL led to the secondary, but non-cytotoxic, effect of rupturing the plasma membrane, which caused the appearance of a sub-population of necrotic cells in a cytometry plot [12].

Data pertaining to SPL immunomodulatory effects in HaCat cells are non-existent but works on other cell lines showed that, in general, SPL extracts had an anti-inflammatory effect in human aortic endothelial, RAW 264.7, and in 3T3-L1 cells, reducing TNF-α, IL-8, and IL-6 expression [32,33,34]. The inflammatory data reported here may be explained by an HaCat-specific sensitivity to plant phenolic compounds, as Pastore, et al. [35] showed that flavonoids, and quercetin in particular, upregulated the IL-6 gene by three-fold.

## 4. Materials and Methods

### 4.1. Chemicals and Raw Materials

The ethanol, formic acid, and chlorogenic acid standard used in this work were of analytical grade and attained from Sigma (Sigma-Aldrich, St. Louis, MO, USA). HPLC and LC–MS solvents were attained pre-prepared from VWR (West Chester, PA, USA). Sweet potato leaves were harvested from an open field cultivation at the end of the production cycle in September 2023 from a local farm in Sanguedo, Vila Nova de Gaia, Portugal. The collected plant material was vacuum sealed and stored at −20 °C until further analysis.

### 4.2. Compound Extraction and Characterization

#### 4.2.1. Extraction

SPL extracts were prepared at 5% (*m*/*v*) using EtOH with 0.1% (*v*/*v*) formic acid and left to react overnight at room temperature. Samples were then centrifuged with supernatants being recovered and solvents evaporated via rotary evaporation (Büchi, Flawil, Switzerland). The resulting powder was then collected and stored under hydroscopic conditions until use.

#### 4.2.2. Phenolic Compounds Fingerprint by uHPLC-DAD

Quantification of the phenolic compounds content was performed using RP-uHPLC-DAD analysis on a Vanquish Core uHPLC system (Thermo Scientific, Waltham, MA, USA), which was equipped with an auto-sampler, pump, column compartment, and diode array detector. Chromatographic separation was achieved using an Acclaim 120 column (Thermo Scientific; C18; 2.1 × 250 mm; particle size 3 µM; pore size 120 Å). The injection volume was set at 1 μL, the column temperature was maintained at 30 °C, and the flow rate was 0.4 mL/min. The elution system for the phenolic compounds consisted of two solvents: solvent A (0.1% formic acid in ultra-pure water, *v*/*v*) and solvent B (0.1% formic acid in acetonitrile). The elution profile was as follows: initial equilibration for 2 min; 0 to 8 min for 28.5% (*v*/*v*) of solvent A with 71.5% (*v*/*v*) solvent B; 8 to 12 min for 28.5% (*v*/*v*) of solvent A with 71.5% (*v*/*v*) solvent B; and 12 to 20 min 100% (*v*/*v*) of solvent A with 0% solvent B. The total acquisition time was 20 min, with a total run time of 22 min. For the quantification of total phenolic compounds by area under the curve (AUC), an authentic standard of chlorogenic acid was used (y = 45.556x + 0.0838; LOD = 0.0037 µg/mL; LOQ = 0.012 µg/mL). The AUC values of the unknown samples were then compared to the calibration curve to estimate the total phenolic content.

#### 4.2.3. High Resolution LC–MS

High-resolution LC–MS analysis was performed according to the method proposed by Monforte et al. [36]. The analysis utilized LC−ESI−UHR−QqTOF−MS (Impact II, Bruker) and was conducted with an UltiMate 3000 Dionex ultra-high-performance liquid chromatography system (UHPLC, Thermo Scientific) coupled to an ultra-high-resolution quadrupole−quadrupole time-of-flight (UHR−QqTOF) mass spectrometer. Data acquisition was performed in negative ionization mode. Metabolite separation was carried out on an Acclaim RSLC 120 C18 column (100 × 2.1 mm, 2.2 μm, Dionex).

The mobile phases consisted of 0.1% formic acid in water (solvent A) and acetonitrile containing 0.1% formic acid (solvent B). The gradient program started at 5% B, increased to 95% B over 7 min, was held at 95% B for 2 min, returned to 5% B within 1 min, and was maintained at 5% B for an additional 5 min. The flow rate was set at 0.25 mL/min, with an injection volume of 5 μL.

For the mass spectrometry, data were collected in positive ionization mode across a mass range of *m*/*z* 20 to 1000. The parameters for the MS analysis were as follows: a capillary voltage of 4.5 kV; a drying gas temperature of 200 °C; a drying gas flow rate of 8.0 L/min; a nebulizing gas pressure of 2 bar; a collision RF of 300 Vpp; a transfer time of 120 μs; and a pre-pulse storage time of 4 μs. Post acquisition, internal mass calibration was performed using sodium formate clusters, delivered via a syringe pump at the start of each chromatographic run.

#### 4.2.4. Data Analysis

LC–MS data were analyzed using MZmine 4.4.3 for mass detection, chromatogram normalization, deconvolution, and alignment. The detected compounds were identified as metabolites based on their retention time (tₒ), accurate molecular mass, predicted molecular formula, and MS/MS spectra by comparison with publicly available databases (e.g., GNPS database and PubChem). Additionally, an MZmine-generated table, containing aligned retention times, mass data (*m*/*z*), and peak area, was further processed using the online workflow Global Natural Products Social Molecular Networking (GNPS). Compound identification was performed using the GNPS match library workflow, with the following parameters: a mass tolerance of 2.0 Da; a fragment tolerance of 0.5 Da; a score threshold of 0.7; and at least 6 matching peaks.

### 4.3. Bacterial Strains and Cell Lines

Microorganisms used in this work were methicillin-sensitive *Staphylococcus aureus* (MSSA, ATCC ATCC25923), methicillin-resistant *S. aureus* (MRSA, ATCC 10145), *Pseudomonas aeruginosa* (*P. aeruginosa*, ATCC 7000699), and *Escherichia coli* (*E. coli*, NCTC 9001). For the antimicrobial testing, all microorganisms were grown in Muller–Hinton Broth (MHB, Biokar, Allone, France) and plated in Plate Count Agar (PCA, Biokar, France). For the antibiofilm assays, E. coli microorganisms were grown in Luria–Bertany broth (LB, Avantor, Carnaxide, Portugal) while Tryptic Soy Broth with 10% (*m*/*v*) glucose was used for the remaining microorganisms. For all assays, test microorganisms were grown at 37 °C.

Human immortalized keratinocytes (HaCat, CLS 300493) were cultured as monolayers using Dulbecco’s Modified Eagle Medium (DMEM) with 4.5 g/L glucose, L-glutamine without pyruvate (ThermoScientific, Waltham, MA, USA) containing 10% fetal bovine serum (ThermoScientific, MA, USA) and 1% (*v*/*v*) Penicillin–-Streptomycin–Fungizone (ThermoScientific, MA, USA). Cells were cultured at 37 °C in a humidified atmosphere of 95% air and 5% CO_2_ and used between passages 33 and 40.

### 4.4. MIC and MBC Determination

SPL extract antimicrobial activity was evaluated as previously described by Costa et al. [37]. Briefly, SPL powder was dissolved into MHB and filtered through a sterile 0.22 µm filter. Following that, a 0.5 MacFarland inoculum of each microorganism was prepared and inoculated in MHB with SPL extract concentrations ranging from 0.1 to 10 mg/mL. In each assay, a positive control (inoculated media) and a negative control (non-inoculated media with SPL extract at 0.1 mg/mL) were also evaluated. The MIC was determined by observing the lowest concentration at which no bacterial growth was visible. For each MIC concentration, 100 µL were plated in PCA; the MBC was defined as the lowest sample concentrations without bacterial growth. All assays were performed in quadruplicate.

### 4.5. Antibiofilm Activity

Evaluation of the anti-biofilm activity was performed as previously described [37]. Briefly, LB broth (*E. coli*) or TSB with 10% glucose and SPL extract at ½ of the MIC were inoculated with test microorganisms at 2% (*v*/*v*) and incubated at 37 °C for 48 h. Following this, the contents of the wells were discarded, washed with sterile PBS, fixed with ethanol, and then stained with crystal violet. Quantification of biofilm formation was performed through a determination of the biomass by measuring the optical density of 630 nm using a microplate reader. All assays were conducted in triplicate; the results are given as a percentage of biofilm formation inhibition.

### 4.6. Cytotoxicity Evaluation

The impact of the samples upon HaCat viability was performed according to the ISO 10993-5:2009 Standard, as previously described by Costa et al. [38], with the following three assays being used to evaluate the extracts impact upon the cell line.

#### 4.6.1. Cellular Metabolism

Cells were grown to 80–90% confluence, detached and seeded at 1 × 10^5^ cells/mL in a 96-well microplate. At 24 h, media were removed and replaced with fresh media supplemented with PSO at concentrations ranging from 1000 to 100 µg/mL. As controls, plain media and media supplemented with DMSO at 30% (*v*/*v*) were used as growth and death controls, respectively. After 24 h of exposure, 100 µL of MTT (solution (0.5 mg/mL) was added to each well; the plates were incubated at 37 °C, in the dark. After 2 h, the MTT solution was removed and 100 µL of DMSO were added. The plates were shaken and protected from light for 10 min. Absorbance was read at 570 nm using a microplate reader (Synergy H1, Biotek Instruments, Winooski, VT, USA). All assays were performed in quadruplicate and results were given in terms of the percentage of cell metabolism inhibition.

#### 4.6.2. Cellular Proliferation

The evaluation of the SPL extracts upon cellular proliferation was performed using the Cyquant Direct Cell Proliferation Assay Kit (Thermofisher, MA, USA). Briefly, cells were grown and seeded, as described in Section 4.6.1, and exposed to extract concentrations without deleterious metabolic effects (both for the samples and controls). After 24 h of exposure, a cell-permeant DNA-binding dye was added to the wells and the plate was re-incubated for 1 h, Fluorescence (Ex: 480 nm; Em: 535 nm) was then measured using a microplate reader. All assays were performed in quadruplicate and results were calculated according to the manufacturer’s instructions.

#### 4.6.3. Cell Viability by Flow Cytometry

The assessment of the impact of extracts upon HaCa cellular viability was performed through flow cytometry, as previously described [39]. Briefly, cells were seeded at 2.5 × 10^5^ cells/mL and, after 24 h, exposed to extract concentrations which presented no deleterious effects upon cellular metabolism. After 24 h, cells were washed with PBS, trypsinized, washed with PBS, and centrifuged at 1500 rpm for 5 min. Following this, cells were treated with the Biolegend FITC Annexin V Apoptosis Detection Kit with 7-AAD (Cat. 640922, San Diego, CA, USA), according to the manufacturer’s instructions. Stained cells were then analyzed via flow cytometry with a BD Accuri™ C6 Flow Cytometer (BD Biosciences, Franklin Lakes, NJ, USA). Results are expressed in percentages of live (Av− 7AAD−), early apoptotic (Av+ 7AAD−), necrotic (Av− 7AAD+), and dead (Av+ 7AAD+) cells. Each sample was evaluated in triplicate.

### 4.7. Inflammatory Potential

The inflammatory potential of the SPL extract upon skin keratinocytes was performed as previously described by Costa et al. [40]. Briefly, HaCat cells were seeded at 2.5 × 10^5^ cells/mL and incubated overnight at 37 °C. Cells were then exposed to SPL at the selected concentrations in the absence (un-stimulated) or presence (stimulated) of lipopolysaccharides from Escherichia coli (serotype O55:B5) (LPS) (Sigma-Aldrich, St. Louis, MO, USA). Plain media were used as the basal control while media with LPS were used as the inflammation control. All assays were performed in quadruplicate. Results were obtained via IL-6 detection and quantification via an ELISA assay using the Human IL-6 Elisa Max Standard Set for IL-6 (BioLegend, San Diego, CA, USA) according to the manufacturer’s instructions. All determinations were performed in quadruplicate and data are given in terms of fold expression increase or decrease relative to basal level expression.

### 4.8. Statistical Analysis

The statistical analysis of the data obtained was performed using IBM SPSS Statistics v21.0.0 (New York, NY, USA). Considering that the data followed a normal distribution, results were analyzed using one-way ANOVA coupled with Turkey’s post hoc test, with differences being considered significant for *p*-values below 0.05.

## 5. Conclusions

In conclusion, this work demonstrates the potential of SPL in skin-related applications. The data obtained reveal a rich phenolic composition, with the identification of compounds directly related to medicinal plants, particularly hyperoside. From a biological standpoint, SPL’s capacity to inhibit microbial growth and biofilm formation of four skin-relevant commensals or pathogenic microorganisms was particularly notable. The only drawback was the pro-inflammatory effect observed in HaCat cells, which need to be addressed in future works. In general, the observed compounds and bioactive properties of SPL suggest the existence of potential for promising applications in skin health, particularly as a natural antimicrobial agent. However, further works are still necessary to understand the full potential of this low-cost extract, particularly regarding its immunomodulation of HaCat cells.

## Figures and Tables

**Table 1:** Compounds identified in the SPL extract by LC–MS.

N°	RT (min)	Area (a.u)	*m*/*z*	Molecular Formula	Metabolite
1	4.81	8.48 × 10^5^	707.18/707.19	C_16_H_18_O_9_	Chlorogenic acid
2	4.9	7.29 × 10^6^	353.08/353.09	C_16_H_18_O_9_	Neochlorogenic Acid
3	8.3531	8.97 × 10^5^	179.03/179.04	C_9_H_8_O_4_	Caffeic acid
4	4.9072	1.05 × 10^6^	431.19/413.19	C_19_H_30_O_8_	3,5,5-Trimethyl-4beta-hydroxy-4-[3-(beta-D-glucopyranosyloxy)-1-butenyl]-2-cyclohexene-1-one
5	9.3774	8.38 × 10^5^	625.14/625.14	C_27_H_30_O_17_	Quercetin-3-O-sophoroside
6	10.9606	5.19 × 10^6^	609.14/609.15	C_27_H_30_O_16_	Rutin
7	11.3332	6.62 × 10^6^	463.09/463.12	C_21_H_20_O_12_	Spiraeoside
8	11.3332	2.50 × 10^6^	927.13/927.18	C_21_H_20_O_12_	Hyperoside
9	12.3578	1.88 × 10^7^	512.12/512.11	C_25_H_24_O_12_	Cynarin
10	16.9211	3.46 × 10^5^	593.13/593.15	C_30_H_26_O_13_	tiliroside
11	17.0142	3.99 × 10^6^	677.15/677.15	C_34_H_30_O_15_	3,4,5-Tricaffeoylquinic acid

**Table 2:** MIC and MBC values attained for the SPL extract against the tested microorganisms. All values in mg/mL. n.d.—not detected.

	MIC	MBC
*P. aeruginosa*	5	5
MRSA	2.5	n.d.
MSSA	5	5
*E. coli*	5	n.d.

## Data Availability

The data presented in this study are available on request from the corresponding author. The data are not publicly available due to confidentiality agreements.

## Abbreviations

The following abbreviations are used in this manuscript:

ATCC	American Type Culture Collection
AUC	Area Under the Curve
CAE	Chlorogenic Acid Equivalents
*E. coli*	*Escherichia coli*
EtOH	Ethanol
GNPS	Global Natural Products Social Molecular Networking
IL-6	Interleukin 6
IL-8	Interleukin 8
LC–MS	Liquid Chromatography–Mass Spectrometry
LOD	Limit of Detection
LOQ	Limit of Quantification
LPS	Lipopolysaccharide
MBC	Minimal Bactericidal Concentration
MCF-10A	Human Mammary Epithelial Cells
MHB	Muller Hinton Broth
MIC	Minimal Inhibitory Concentration
MRSA	Methicillin Resistant *Staphylococcus aureus*
MSSA	Methicillin Sensitive *Staphylococcus aureus*
NCTC	National Collection of Type Cultures
*P. aeruginosa*	*Pseudomonas aeruginosa*
PCA	Plate Count Agar
RT	Retention Time
RP-uHPLC-DAD	Reverse-Phase Ultra-High-Performance Liquid Chromatography with Diode Array Detection
SPL	Sweet Potato Leaves
TFC	Total Flavonoid Content
TPC	Total Phenolic Content
TNF-α	Tumor Necrosis Factor Alpha
uHPLC-DAD	Ultra-High Performance Liquid Chromatography with Diode Array Detection
UHR−QqTOF	Ultra-High-Resolution Quadrupole−Quadrupole Time of Flight
TLA	Three Letter Acronym
LD	Linear Dichroism
